# Lumbar muscle and vertebral bodies segmentation of chemical shift encoding-based water-fat MRI: the reference database MyoSegmenTUM spine

**DOI:** 10.1186/s12891-019-2528-x

**Published:** 2019-04-09

**Authors:** Egon Burian, Alexander Rohrmeier, Sarah Schlaeger, Michael Dieckmeyer, Maximilian N. Diefenbach, Jan Syväri, Elisabeth Klupp, Dominik Weidlich, Claus Zimmer, Ernst J. Rummeny, Dimitrios C. Karampinos, Jan S. Kirschke, Thomas Baum

**Affiliations:** 1 Department of Diagnostic and Interventional Neuroradiology, Klinikum rechts der Isar, Technische Universität München, Munich, Germany; 2 Department of Diagnostic and Interventional Radiology, Klinikum rechts der Isar, Technische Universität München, Munich, Germany

**Keywords:** Magnetic resonance imaging, Proton density fat fraction, Lumbar spine, Muscle, Bone marrow, Segmentation

## Abstract

**Background:**

Magnetic resonance imaging (MRI) is the modality of choice for diagnosing and monitoring muscular tissue pathologies and bone marrow alterations in the context of lower back pain, neuromuscular diseases and osteoporosis. Chemical shift encoding-based water-fat MRI allows for reliable determination of proton density fat fraction (PDFF) of the muscle and bone marrow. Prior to quantitative data extraction, segmentation of the examined structures is needed. Performed manually, the segmentation process is time consuming and therefore limiting the clinical applicability. Thus, the development of automated segmentation algorithms is an ongoing research focus.

**Construction and content:**

This database provides ground truth data which may help to develop and test automatic lumbar muscle and vertebra segmentation algorithms. Lumbar muscle groups and vertebral bodies (L1 to L5) were manually segmented in chemical shift encoding-based water-fat MRI and made publically available in the database MyoSegmenTUM. The database consists of water, fat and PDFF images with corresponding segmentation masks for lumbar muscle groups (right/left erector spinae and psoas muscles, respectively) and lumbar vertebral bodies 1–5 of 54 healthy Caucasian subjects. The database is freely accessible online at https://osf.io/3j54b/?view_only=f5089274d4a449cda2fef1d2df0ecc56.

**Conclusion:**

A development and testing of segmentation algorithms based on this database may allow the use of quantitative MRI in clinical routine.

## Background

Morphology-based magnetic resonance imaging (MRI) is clinically used to identify and monitor pathological changes of the spine in many diseases (e.g. osteoporosis or lower back pain) [1–3]. Quantitative MR including magnetic resonance spectroscopy and chemical shift encoding-based water-fat MRI allows for reliable determination of muscle and bone marrow fat composition [4, 5]. It can be used for the quantitative assessment of muscular tissue composition in neuromuscular disorders and their longitudinal monitoring [6, 7]. It was reported that intramuscular fat is increased in patients with neuromuscular diseases and lower back pain [3, 7–10]. Bone marrow malignancies, osteoporosis and distinction of the fractures are also common fields of application for quantitative MRI [11–13]. Compared to quantitative MRI, morphology-based MRI with the subsequent qualitative analysis and generation of semiquantitative parameters is limited in this context considering interrater objectivity and experience-based follow up evaluation [14].

Proton density fat fraction (PDFF) can be calculated based on chemical shift encoding-based water-fat MRI [15, 16]. However, up to now a manually segmentation process is needed to extract PDFF values from the lumbar muscles and the vertebral bone marrow. This is time consuming and therefore limiting the clinical applicability. Clinicians as well as scientists would strongly benefit from automated segmentation methods. Despite some recent developments in (semi-) automated segmentation techniques in different imaging disciplines their transfer into clinical routine is still challenging [17–19]. Previous work reported possibilities for muscle and vertebrae segmentation in MR images [20–23]. These algorithms require large datasets to test their performance and to further develop them. However, the public availability of manually segmented datasets of lumbar muscle groups and vertebrae as ground truth are limited.

Therefore, the purpose of this work was to provide a ground truth database with quantitative chemical shift encoding-based water-fat MR images and segmentations of the lumbar muscle groups (right/left erector spinae and psoas muscles, respectively) and the lumbar vertebral bodies (L1 to L5).

## Construction and content

### Subjects

The database contains 54 MRI datasets of healthy, Caucasian volunteers (15 males, 39 female, age: 51.6 ± 16.7 years).

### MR imaging

Fifty-four healthy volunteers underwent MRI on a 3 T system (Ingenia, Philips Healthcare, Best, Netherlands) using a whole-body coil, the built-in 12-channel posterior coil and a 16-channel anterior coil. Subjects were positioned head-first in a supine position. Two different sequences were used for imaging the lumbar muscles and vertebral bodies. The conducted scanning protocol included dedicated scanning parameters, which are shown in Table 1.

**Table 1:** Scan parameters of the axially- and sagittally-prescribed sequence

	Axially-prescribed six-echo 3D spoiled gradient- echo sequence	Sagittally-prescribed eight-echo 3D spoiled gradient-echo sequence
TR/ TE1/ ∆TE	6.4/1.1/0.8 ms	11/1.4/1.1 ms
Flip angle	3°	3°
Bandwith	2484 Hz/pixel	1527 Hz/pixel
Acquisition matrix	68 × 150	124 × 121
Field of view (FOV)	220 × 401 × 252 mm^3^	220 × 220 × 80 mm^3^
Acquisition voxel size	3.2 × 2.0 × 4.0 mm^3^	1.8 × 1.8 × 4.0 mm^3^
Frequency direction	L/R	A/P
Scan time	1 min and 25 s	1 min and 17 s

The acquired data were analyzed using the mDIXON fat quantification method provided by the manufacturer. A complex-based water-fat decomposition was performed using a single T_2_* correction and a pre-calibrated fat spectrum accounting for the presence of the multiple peaks in the fat spectrum [24]. A seven-peak fat spectrum model was employed. The imaging-based proton density fat fraction (PDFF) map was computed as the ratio of the fat signal over the sum of fat and water signals, described previously by Schlaeger et al. [25, 26].

Axial and sagittal water, fat and PDFF images were stored as separate datasets for each subject as a *.dcm file.

### MR image segmentation

The subsequent segmentation of the computed PDFF was performed manually using the open access image viewer software MITK (Medical Imaging Interaction Toolkit, Heidelberg, Germany). All segmentations were performed by a board certified radiologist.

**Fig. 1:**
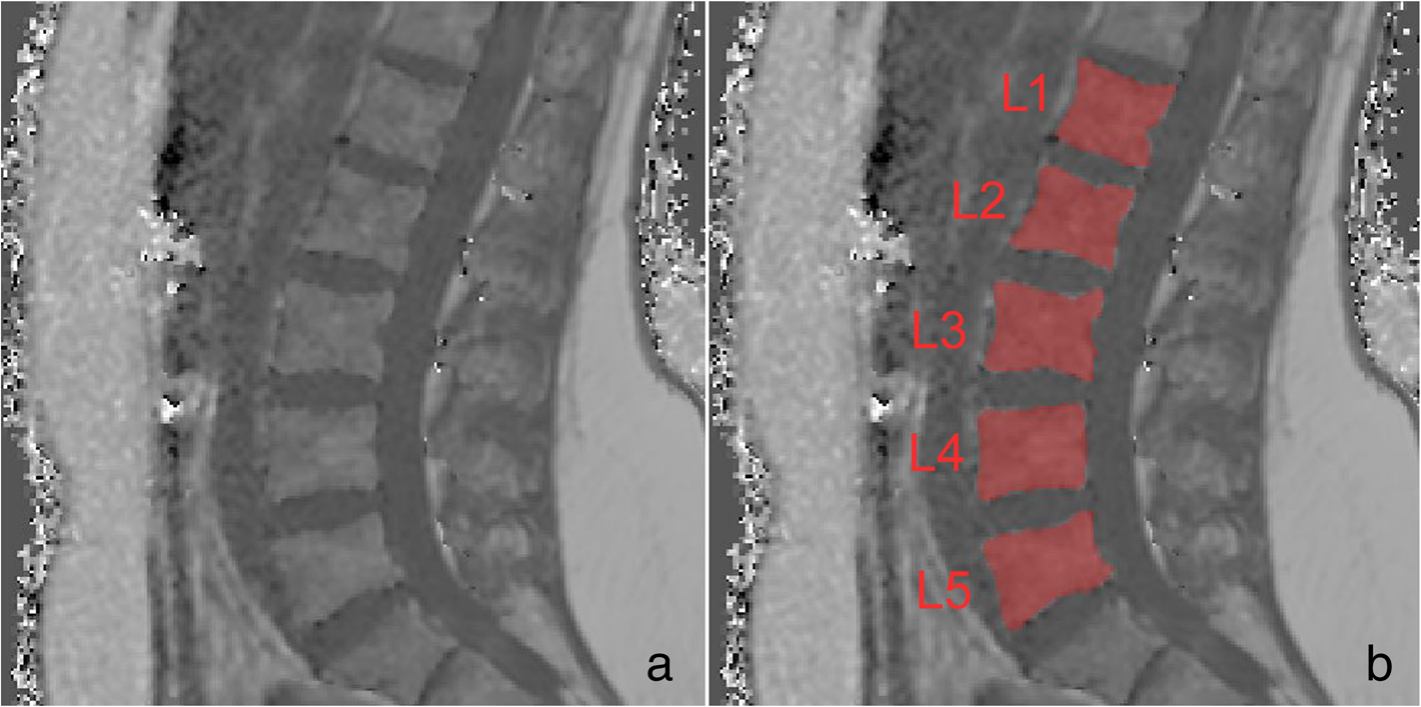
(**a**) PDFF map of a 28-year-old male with representative segmentation of the vertebral bodies L1 to L5 (**b**)

The vertebral bodies L1 to L5 were segmented in the sagittal PDFF maps excluding the posterior elements (Fig. 1).

**Fig. 2:**
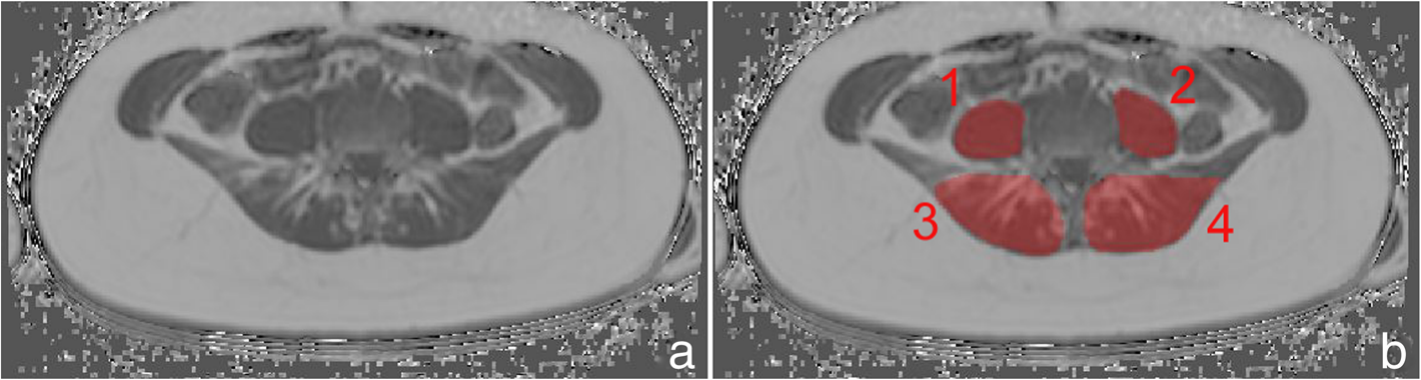
(**a**) PDFF map of a 28-year-old male with representative segmentation of the following muscle groups (**b**): (1) right psoas muscle, (2) left psoas muscle, (3) right erector spinae muscle, and (4) left erector spinae muscle

The axial PDFF maps were used to separately segment the erector spinae and the psoas muscle on both sides from the cranial part of L2 to caudal part of L5. Figure 2 exemplarily shows the depicted ROI in the corresponding muscles.

PDFF of each vertebral body (L1 to L5) and muscle group (right/left erector spinae and psoas muscle, respectively) were extracted. The segmentations of each vertebral body and muscle group is available as a binary mask. Each mask was stored as a separate *.mha file.

## Utility and discussion

### Database availability

The database is available online at https://osf.io/3j54b/?view_only=f5089274d4a449cda2fef1d2df0ecc56. Axial and sagittal water, fat and PDFF images are deposited as separate datasets for each subject as a *.dcm file. The segmentation masks of each vertebra (L1 to L5) and muscle group were deposited as *.mha files.

### Subjects’ characteristics

Datasets of subjects and corresponding segmentation masks are labeled with the same subject ID (1 to 54). Masks were labeled as L1 to L5 or muscle group (right erector spinae muscle, left erector spinae muscle, right psoas muscle, and left psoas muscle, respectively).

Subject characteristics (sex, age [years], weight [kg], height [m] and body mass index (BMI) [kg/m^2^]) and PDFF values [%] of the vertebral bone marrow (L1 to L5) and the lumbar muscle groups (right/left erector spinae and psoas muscle, respectively) are listed in Table 2. Segmentation of the paraspinal muscles and the lumbar vertebrae (L1 to L5) amounted 50 min and 1:40 h for each subject, respectively.

**Table 2:** Subject characteristics and PDFF values of the lumbar muscle groups (right/left erector spinae and psoas muscle, respectively) and the vertebral bone marrow (L1 to L5)

ID	sex	age [y]	height [m]	weight [kg]	BMI [kg/m^2^]	PDFF erector spinae muscle left [%]	PDFF erector spinae muscle right [%]	PDFF psoas muscle left [%]	PDFF psoas muscle right [%]	PDFF L1 [%]	PDFF L2 [%]	PDFF L3 [%]	PDFF L4 [%]	PDFF L5 [%]
1	female	39	1.64	69	25.7	8.0	10.3	2.9	2.7	22.7	22.3	22.4	23.6	23.8
2	female	25	1.69	75	26.3	11.2	9.1	2.8	1.5	21.6	22.1	24.0	24.4	25.6
3	male	31	1.88	87	24.6	5.8	7.9	4.8	2.3	18.6	18.2	17.7	20.8	19.9
4	male	41	1.75	79	25.8	7.8	8.9	5.4	3.6	23.9	26.5	26.7	27.8	27.5
5	male	28	1.71	73	25.0	10.6	9.2	4.1	2.1	25.5	26.1	26.5	27.7	30.4
6	female	28	1.81	93	28.4	7.7	9.5	5.2	3.8	41.1	42.7	42.4	42.3	45.1
7	female	22	1.71	77	26.3	14.4	14.2	5.7	5.5	23.0	24.6	24.1	25.6	27.1
8	female	26	1.66	72	26.1	12.2	13.7	4.7	4.5	22.9	25.7	27.6	28.4	28.4
9	female	41	1.64	76	28.3	15.7	15.6	8.8	5.9	33.7	38.3	39.8	39.7	42.3
10	female	38	1.72	70	23.7	11.9	12.3	2.2	2.3	17.2	19.2	20.3	20.7	23.3
11	female	33	1.64	70	26.0	6.4	4.1	7.2	9.0	27.0	26.8	28.4	32.5	32.4
12	male	32	1.79	104	32.5	8.3	8.5	4.1	3.9	34.4	34.1	35.4	37.9	41.3
13	female	21	1.63	64	24.1	9.3	8.8	3.5	4.5	30.4	33.5	35.6	36.7	38.7
14	male	27	1.90	108	29.9	7.7	7.5	5.2	4.5	36.5	34.6	37.2	37.6	43.8
15	male	24	2.00	105	26.3	11.3	8.7	6.2	4.3	27.8	28.8	29.0	30.6	33.3
16	female	27	1.79	79	24.7	8.9	9.3	6.6	4.8	16.7	17.8	19.7	19.9	19.2
17	male	30	1.78	96	30.3	14.4	14.9	5.5	4.8	34.5	36.3	38.6	41.0	45.5
18	male	37	1.86	95	27.5	12.1	12.4	5.7	5.6	42.3	40.9	40.8	43.6	47.2
19	male	34	1.90	112	31.0	8.5	8.1	4.5	3.8	29.9	25.3	30.1	39.5	46.1
20	female	24	1.74	73	24.1	11.8	12.8	6.0	4.1	42.8	40.3	47.4	49.0	49.8
21	female	42	1.58	68	27.2	13.8	13.6	7.0	5.6	28.9	30.0	29.6	31.9	35.2
22	male	32	1.74	94	31.0	9.3	8.4	5.9	4.2	32.9	34.5	35.0	36.0	38.2
23	male	27	1.83	92	27.5	14.1	15.2	5.8	6.1	27.6	29.8	32.0	33.6	34.8
24	female	32	1.59	69	27.3	13.2	14.7	6.4	5.6	20.5	23.7	24.6	25.9	26.2
25	male	33	1.75	79	25.8	9.0	8.5	6.4	5.2	37.1	39.7	43.3	47.8	45.3
26	male	33	1.78	77	24.3	9.2	7.2	1.9	1.6	31.7	29.3	30.7	31.8	34.0
27	female	26	1.71	81	27.7	14.6	14.3	5.8	5.9	23.1	24.5	23.9	25.8	26.5
28	male	26	1.76	76	24.4	7.2	7.5	5.8	5.5	31.4	31.5	35.0	34.5	35.8
29	female	25	1.63	65	24.5	14.5	13.4	6.5	4.9	22.0	21.7	21.9	25.3	25.9
30	male	23	1.87	86	24.6	10.0	9.6	1.6	0.2	18.6	19.0	20.8	22.4	21.7
31	female	61	1.68	72	25.5	17.3	18.3	0.2	3.8	59.2	54.1	55.3	62.1	64.9
32	female	61	1.59	52	20.6	18.0	15.5	2.6	4.2	28.6	29.9	28.0	35.3	31.7
33	female	78	1.59	77	30.5	36.9	36.5	2.9	9.9	32.65	42.17	50.45	55.94	58.86
34	female	64	1.59	57	22.5	20.6	23.0	−0.5	1.3	46.7	44.6	45.5	56.9	62.2
35	female	54	1.65	82	30.1	17.7	18.5	−0.4	1.7	33.7	34.5	35.5	37.2	38.7
36	female	60	1.65	75	27.5	22.2	24.2	6.5	5.8	50.9	53.5	55.5	45.3	62.9
37	female	67	1.65	70	25.7	16.6	19.8	10.6	8.9	39.8	39.8	42.3	44.6	55.7
38	female	59	1.74	79	26.1	16.9	19.6	0.5	2.1	49.0	49.5	51.8	56.1	62.0
39	female	63	1.64	95	35.3	32.0	26.2	8.7	7.8	48.7	49.9	51.9	59.7	62.7
40	female	71	1.63	60	22.6	25.5	21.6	1.5	1.3	45.7	53.5	50.5	50.7	50.4
41	female	58	1.65	65	23.9	20.7	20.4	3.5	−0.3	41.1	40.3	43.7	45.6	45.9
42	female	59	1.67	84	30.1	18.6	20.5	1.8	2.8	35.7	36.0	36.2	49.1	54.0
43	female	60	1.68	76	26.9	21.6	23.4	9.3	9.4	43.0	40.8	47.6	47.9	54.1
44	female	63	1.77	98	31.3	20.9	24.4	8.5	6.7	49.0	50.5	54.0	60.6	62.5
45	female	58	1.68	61	21.6	22.0	27.7	4.9	3.5	49.7	41.0	41.4	49.9	51.4
46	female	74	1.71	56	19.2	25.9	25.2	4.4	4.5	40.1	41.3	41.9	42.2	49.1
47	female	61	1.65	65	23.9	32.0	33.2	5.4	5.0	52.6	54.5	51.0	51.8	51.5
48	female	68	1.71	84	28.7	45.8	41.5	9.3	9.9	58.2	58.0	60.4	58.3	62.9
49	female	71	1.60	53	20.7	27.3	26.3	7.2	2.4	37.5	32.6	39.4	41.2	38.5
50	female	68	1.67	65	23.3	32.3	34.2	5.0	8.5	43.7	44.0	49.0	50.6	59.9
51	female	59	1.58	47	18.8	28.6	32.7	5.8	6.1	58.3	58.5	60.3	64.3	67.8
52	female	69	1.64	73	27.1	32.3	28.1	7.4	8.3	39.3	37.0	40.0	41.3	43.9
53	female	55	1.68	69	24.4	17.5	18.5	2.6	5.9	37.5	41.1	43.0	46.0	44.9
54	female	55	1.60	77	30.1	16.3	16.7	1.3	3.1	30.8	29.3	31.4	26.7	35.7

### Discussion

Recent and future trends of imaging automatization are and surely will be influencing many aspects of radiological diagnostics now and in the future. Subspecialties as neuroradiology, breast imaging, musculoskeletal radiology and oncological imaging are affected regarding (semi-) automated initial diagnostic finding, disease monitoring and report classification [18, 27–29]. Our aim with offering a database for manually segmented lumbar muscles and vertebral bodies (L1 to L5) in MR images of 54 healthy volunteers is to provide ground truth data which supports testing and refining newly developed computer vision or machine learning algorithms for automatic lumbar muscle and spine segmentation. The present database offers access to water, fat and PDFF maps.

**Fig. 3:**
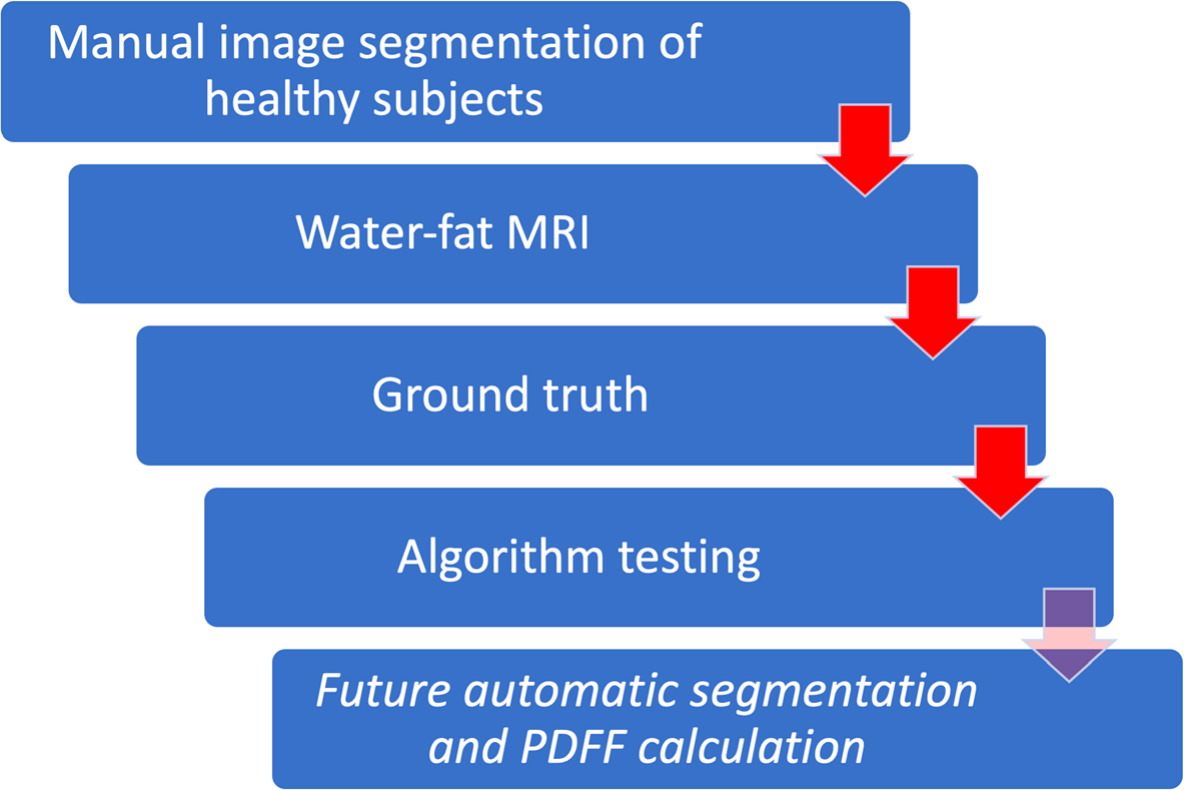
Flowchart with purpose and workflow of the study

With the extractable volume and PDFF values for the psoas and erector spinae muscles and the vertebral bodies L1 to L5, we elucidate the use of quantitative MRI in spine and muscle imaging and offer a ground truth dataset for automatic algorithm testing purposes. Figure 1 exemplarily illustrates the PDFF map of the vertebral bodies L1 to L5 and Fig. 2 shows a PDFF map for the respective muscle groups. The presented data is in line with the studies of Schlaeger et al. and Baum et al. and could help to develop more efficient ways of segmenting musculoskeletal structures [25, 30]. This is necessary to enable scientists and clinicians to perform data processing and analysis in an economic way. Additionally, the described methodical aspects are of great importance in order to being able to transfer automatized quantitative MRI into clinical routine by promoting computational segmenting and analyzing PDFF maps. In a further step the engineering of a fully automatic diagnosis finding due to pattern based deep learning could be possible and highly beneficial to diagnostic accuracy, interreader reliability and longitudinal comparability (Fig. 3) [31]. As recently shown in skin cancer diagnostics huge amounts of datasets are needed to constitute an algorithm quality that can outperform a specialist’s evaluation [28]. The amount of data generated with each qualitative respectively quantitative MRI scan delineates information which needs to be assessed carefully and precisely. This sets the high standards to every algorithm competing with specialists’ knowledge and skills. Complex disease entities like neuromuscular disorders, osteoporosis or lower back pain can be represented in different anatomical structures from very subtle bony or muscular alterations to striking pathologic imaging findings in cases of fractures or muscle atrophy [2, 5, 32, 33].

This database should be considered as one contribution to an ongoing process of rationalization and automatization. The limitation of the present database is the inclusion of healthy subjects only. Thus, this database has to be extended by diseased subjects in the future.

## Conclusion

We present the database MyoSegmenTUM Spine with manually segmented lumbar muscle groups and vertebral bodies in MR images of 54 healthy volunteers together with corresponding manual segmentation masks. The data can be used as a training and test datasets for the development of automatic lumbar muscle and spine segmentation algorithms. These algorithms are highly needed to promote and accelerate the wide spread clinical implementation of quantitative muscle MRI for diagnosis of muscle and vertebral pathologies.

## Abbreviations

A/P: Anterior/posterior
FOV: Field of view
L/R: Left/right
L1, L2 etc.: Lumbar vertebrae 1, lumbar vertebrae 2 etc.
MRI: Magnetic Resonance Imaging
PDFF: Proton density fat fraction
TE: Echo time
TR: Repetition time
